# Algorithmic Profiling of Job Seekers in Austria: How Austerity Politics Are Made Effective

**DOI:** 10.3389/fdata.2020.00005

**Published:** 2020-02-21

**Authors:** Doris Allhutter, Florian Cech, Fabian Fischer, Gabriel Grill, Astrid Mager

**Affiliations:** ^1^Institute of Technology Assessment, Austrian Academy of Sciences, Vienna, Austria; ^2^Centre for Informatics and Society, Faculty for Informatics, Vienna University of Technology (TU Wien), Vienna, Austria; ^3^School of Information, University of Michigan, Ann Arbor, MI, United States

**Keywords:** algorithmic profiling, public employment service, Austria, big data, critical data studies, qualitative research, coproduction, austerity politics

## Abstract

As of 2020, the Public Employment Service Austria (AMS) makes use of algorithmic profiling of job seekers to increase the efficiency of its counseling process and the effectiveness of active labor market programs. Based on a statistical model of job seekers' prospects on the labor market, the system—that has become known as the AMS algorithm—is designed to classify clients of the AMS into three categories: those with high chances to find a job within half a year, those with mediocre prospects on the job market, and those clients with a bad outlook of employment in the next 2 years. Depending on the category a particular job seeker is classified under, they will be offered differing support in (re)entering the labor market. Based in science and technology studies, critical data studies and research on fairness, accountability and transparency of algorithmic systems, this paper examines the inherent politics of the AMS algorithm. An in-depth analysis of relevant technical documentation and policy documents investigates crucial conceptual, technical, and social implications of the system. The analysis shows how the design of the algorithm is influenced by technical affordances, but also by social values, norms, and goals. A discussion of the tensions, challenges and possible biases that the system entails calls into question the objectivity and neutrality of data claims and of high hopes pinned on evidence-based decision-making. In this way, the paper sheds light on the coproduction of (semi)automated managerial practices in employment agencies and the framing of unemployment under austerity politics.

## 1. Introduction

In the last 30 years, social security systems across Europe went through a paradigmatic transformation of the welfare state to an “enabling state” (Deeming and Smyth, 2015). Under growing unemployment and austerity measures, “social policies are characterized by a shift toward activation regimes, which aim at mobilizing citizen's self-responsibility” (Glinsner et al., 2018, p. 1) and supplement or replace the rights-based access to welfare. Employment administrations have been transformed into consumer-oriented service providers (Penz et al., 2017). They are governed by a managerialism that wants “to save money, increase efficiency, and simultaneously oblige public bureaucracies to act more responsively toward their citizen-users” (Pollitt and Bouckaert, 2011, p. 6). The Public Employment Service Austria (Arbeitsmarktservice, AMS) has been remodeled to a semi-autonomous agency between the mid-1990s and the early 2000s (Ludwig-Mayerhofer and Wroblewski, 2004). As Penz et al. (2017, p. 549) study on the entrepreneurial governance of employment agencies in Austria, Germany and Switzerland shows their “overarching objective […]—and the most important performance indicator—consists in the fast (and sustainable) reintegration of jobseekers into the labor market.”

In October 2018, the AMS announced plans to roll out an algorithmic profiling system. Based on a statistical model of job seekers' prospects on the job market, the system is designed to classify job seekers into three categories:

- Group A: High prospects in the short term
- Group C: Low prospects in the long-term
- Group B: Mediocre prospects, meaning job seekers not part of groups A or C.

Depending on the category a particular job seeker falls under, they will be offered differing support in (re-)entering the labor market. Group A consists of customers with a probability of more than 66% to find employment for at least 3 months within the next 7 months. This segment of clients will receive less support through active labor market programs as it is assumed they will likely find employment without further training. Group C comprises customers with a probability of <25% to be employed for at least 6 months within the next 2 years. This segment is also supposed to receive less support measures from the AMS and is to be assigned to an external institution for supervision instead. The AMS justifies this decision with their experience that expensive active labor market programs do not significantly increase the hiring chances of these two groups. In turn, high investments in groups A and C are not deemed cost effective. The financial focus therefore will be put on group B comprising all customers not part of the aforementioned groups.

The semi-automated classification is explicitly introduced to distribute scarce resources in the active labor market program in an efficient way, i.e., considering the predicted difference of how fast and how sustainable job seekers can be reintegrated into the labor market. The system uses data referring to the individual job seeker's employment history, but also predicts their reintegration chances by taking into account existing inequalities in the labor market that are based on e.g., gender, age, citizenship, and health conditions. After a test phase in 2019 the system will be introduced nation-wide in 2020. The project—commonly referred to as the “AMS algorithm”—has created a heated public debate. Criticism pointed to a lack of transparency, missing possibilities to remedy decisions for AMS customers, the use of sensitive information, possible unintended consequences, such as discrimination, misinterpretation and stigmatization, as well as its potential for reinforcing and amplifying inequality on the labor market. In particular, the inclusion of gender as a variable raised public concerns about gender discrimination. The illustrative model that is part of the documentation lists “female” as being detrimental to the chances of labor market integration reducing the total score of this group (−0.14). Additionally, the variable “obligations of care” was noted to only apply to women. This highly controversial choice was justified by the president of the AMS with the minimal impact of care obligations on men's job prospects as observed empirically. According to the AMS, the system captures the “harsh reality” of the labor market by making realistic predictions for job seekers belonging to disadvantaged groups (Buchinger, 2019; Volksanwaltschaft Österreich, 2019). This statement insists on the objectivity of the data claims and correlations that the system produces.

This paper problematizes claims that the profiling system is neutral and objective. We argue that the system merely approximates the labor market's current state based on chosen data sources, attributes and methods reflecting value-laden choices of system designers, entrepreneurial practices of employment agencies and changes of social security systems that enact the activation paradigm (Penz et al., 2017). In this vein, we trace how the rhetoric of the system's *objectivity* and *precision* is *coproduced* (Jasanoff, 2004) with practices of increasing the *efficiency* of public employment bureaucracies and the need for *effective* resource allocation in the active labor market programs.

Based in science and technology studies (Rose, 1998; Bowker, 2005; Jasanoff, 2006b, 2017), our analysis uses insights from critical data studies (Boyd and Crawford, 2012; O'Neil, 2016; Rieder and Simon, 2016; Noble, 2018) and research on fairness, accountability and transparency in algorithmic systems (Sandvig et al., 2014; Crawford et al., 2019) to examine the inherent politics of the AMS profiling system. An in-depth analysis of relevant technical documentation and policy documents sheds light on a number of research questions that capture crucial conceptual, technical and social implications of the systems. On a conceptual level, we ask how the (semi-)automated profiling of job seekers frames the problem of unemployment and employability and how this framing is enacted by the socio-technical specification of the system. With regard to the technical specification of the system, we ask what data and personal attributes of job seekers are considered in the statistical model, which ones are neglected and what kind of biases this may entail. What performance measures are used to document the reliability of the system and what do they imply? The system's implementation within the organizational practices of the AMS has extensive social implications. What does it imply for the distribution of agency and accountability between the system and the case workers? How have issues of transparency and accountability of the system been handled during the development and test phases?

The paper proceeds with a description of our theoretical and methodical approach as well as the material that this study is based on. The third section engages in an in-depth system analysis of the AMS algorithm and is structured as follows: It delivers a reconstruction of the technical workings of the system and discusses issues of bias and discrimination (section 3.1); it then investigates some of the conceptual principles that guided its implementation (section 3.2) and the way the available documentation communicates the quality of the overall system to the public and oversight bodies (section 3.3); finally, it discusses the system's integration into the counseling practices of the AMS (section 3.4). The fourth section (section 4) provides a discussion of the results of our study and considers broader issues of transparency and accountability. The final section draws conclusions on the coproduction of (semi-)automated managerial practices in public employment agencies and the framing of unemployment under austerity politics.

## 2. Theoretical and Methodical Framework

### 2.1. Coproducing Austerity Politics and Algorithmic Classifications

Empirical studies of changes in the AMS have focused on the employment agency's entrepreneurial remodeling and the neoliberal turn that adopts the “market” as “the organizing principle of state, politics and society” (Penz et al., 2017, p. 541) and made effectiveness and efficiency guiding principles in the agency's practices (Ludwig-Mayerhofer and Wroblewski, 2004). This research provides a rich resource for analyzing the emergent socio-technical implications of introducing an algorithmic profiling system that is supposed to raise the effectiveness of active labor market programs. The production of statistical predictions, and thus, recommendations on which customers to award participation in a program or what quality of program to grant them, needs to be studied in light of the neoliberal shift in social security systems. Like Switzerland and Germany, Austria turned to activation policies in the 1990s (Penz et al., 2017, p. 548). Following Serrano Pascual (2007, p. 14) the activation paradigm is characterized by its “individualized approach” that aims at changing the behavior, motivation and competences of job seekers instead of taking structural measures against unemployment. The relation between the state and the citizen is based on a contract that customers have to sign and by which they agree to fulfill certain obligations in order to obtain benefits. Participation in active labor market measures is one of them. The framing of citizens as customers also includes a “moral contract” that constitutes benefit recipients as a burden to society and morally obliged to work (Penz et al., 2017, p. 544). In the “logic of workfare” wage labor becomes a necessary precondition for the social participation and autonomy of individuals.

This paper focuses in on the tensions and ambivalences that the introduction of a data-based decision-support system entails. The AMS algorithm creates a particular kind of knowledge that reflects conjunctions of normative interests and data, both on individual and aggregate (group) levels. Social studies of science and technology (STS) remind us that technology “both embeds and is embedded in social practices, identities, norms, conventions, discourses, instruments, and institutions” (Jasanoff, 2006b, p. 3). The notion of coproduction of science, technology and social order provides an interpretive framework that explores how “knowledge-making” is entangled with practices of governance and “how practices of governance influence the making and use of knowledge” (Jasanoff, 2006b, p. 3). Technical artifacts, such as the AMS algorithm as well as the knowledge or “facts” they establish are interwoven with values, discourses and political trajectories in ways that demand sustained critical inquiry. They are ordering instruments that “stabilize both what we know and how we know it” (Jasanoff, 2006c, p. 39). As Jasanoff (2017, p. 2) points out in her paper on the political force of data compilations, the making of data claims or correlations relies on particular epistemic presumptions that shape their making in the first place:

“[T]he aggregated incidences represented by a data set have to have meaning, as standing for a classifiable, coherent phenomenon in the world. At the same time, for data to have an impact on law and policy, information must be seen as actionable, that is, numbers or other quantitative representations, such as charts and graphs, must show people both something actual and something that begs to be investigated, explained, or solved. In short, if a data set is to elicit a social response, knowledge of something that matters and principles for understanding why it matters must be generated together, or coproduced.”

This quote points to several issues that are crucial for our analysis of the AMS algorithm. First, the classification frames the phenomenon of unemployment and the prospects for efficient and sustainable reintegration to the labor market in terms of personal characteristics of job seekers and group characteristics based on their gender, age group, dis/ability status, and ethnic descent. This framing individualizes the problem and excludes macro level perspectives on unemployment. Second, the technical system and the data that it is built on does not just reflect an objective, data-based way of modeling employment prospects. The algorithm is coproduced with the objectives and discourses surrounding its introduction, with public discourses on unemployment and the unemployed, and the wider political framework of social security policies that it is embedded in and that it is supposed to stabilize. Of equal importance are the technical concepts and methods that build the algorithm and make it intelligible to the involved actors, e.g., the performance metrics that symbolize its quality and objectivity. All of these normative enactments are entangled in multi-layered ways.

The coproductionist vantage point of this paper builds on findings from critical data studies and research in fairness, accountability and transparency (FAT) in algorithmic systems. One of the main targets of introducing algorithmic systems in private and public organizations is to raise the efficiency of decision-making processes. Moreover, (semi-)automated systems are often argued to produce more objective and unbiased outcomes than humans who may (willingly or out of ignorance) make decisions that privilege certain groups while discriminating against other groups. However, critical data studies and FAT research have given detailed accounts of how algorithmic systems (re-)produce structural discrimination, such as classism, racism, ableism, genderism, and heteronormativity and shows how statistical modeling, machine learning and artificial intelligence may objectify discriminatory outcomes in domains, such as education, employment, health, banking, policing, and counter-terrorism (for a collection of research see Crawford et al., 2019). Algorithmic bias is a socio-technical phenomenon that may emerge from bias in the training data of systems, their classification or the models that are built from this data (see e.g., Sandvig et al., 2014; Buolamwini and Gebru, 2018). It can also be located in the implicit epistemic norms of computational approaches, concepts, methods and tools (Dobbe et al., 2018; Allhutter, 2019; Selbst et al., 2019).

A coproductionist perspective needs to acknowledge that statistical classification co-constitutes the entities that it differentiates between. When a system differentiates between groups of people for a particular purpose based on their gender, ethnicity, age, dis/ability, or socioeconomic status, it re-iterates and coproduces meanings of what these categories imply in a certain context (Allhutter, 2019). While these categories are intersectional and contextually contingent, algorithmic systems frequently model them as dichotomous and essentialist attributes of people (see e.g., Hu and Kohler-Hausmann, 2020).

Introducing (semi-)automated systems in public domains raises profound issues with regard to their transparency and accountability. As our analysis will show, an algorithmic system is not merely a passive technical tool but gains agency through everyday use. In her book “Automating Inequality” Eubanks (2018) demonstrates how algorithmic technologies used in US social service programs classify and manage the poor under austerity politics, instead of tackling poverty itself (see also Gandy, 1993). Gangadharan and Niklas (2019) suggest not losing sight of systematic forms of injustice and discrimination that are not technologically mediated. They point to already existing problems in the architecture of the welfare state: “Welfare recipients who face welfare bureaucracy already feel powerless, and automation may worsen their powerlessness” (p. 890). Systems, such as the AMS algorithm do not just supplement or enhance current counseling processes but they come with subtle and not so subtle changes in the practices of employment agencies. Open remains the question of who is to be held accountable for discriminatory outcomes that are triggered by algorithmic recommendation, and what procedures of public checks and balances are available to affected citizens, but also society as a whole.

### 2.2. Study and Methods

To reconstruct and examine both the social and technical shaping of the AMS algorithm in the context of larger socio-political developments we employ a qualitative document analysis in the tradition of constructionist research (Bowen, 2009; Silverman, 2015). To gain an overview of public and policy debates dealing with the introduction of the algorithm, we collected an extensive amount of documents ranging from (online) newspapers, workshop presentation slides, studies, technical reports and policy documents, to reports by and to public oversight bodies (from the period of December 2017 until August 2019). We selected a core body of documents for a closer analysis: policy documents released by governmental institutions, concept papers by Synthesis GmbH, the company contracted with the development of the algorithm, and by the AMS itself, as well as reports and evaluations by oversight bodies. These key documents enable us to follow the algorithm in-the-making, institutional argumentations to legitimize the introduction of the algorithm, and roles and functions the algorithm is supposed to play in the future AMS practice. Accordingly, we will not try to reveal a hidden “truth” of the algorithm, but rather examine how the algorithm was rhetorically framed, legitimized and technically shaped in the months before and during its test phase, which started in November 2018. A brief introduction to the selected documents^1^ gives a first insight into their specificities:

1. “Report of the Court of Auditors. Public Employment Service” (COURT OF AUD: December 2017):^2^ This report features the AMS algorithm as one of the planned projects to raise the efficiency and effectivity of the AMS. It describes the initial goals and expected achievements of the algorithm and reflects different facets of the neoliberal shift (Rechnungshof, 2017).
2. “The AMS-Employment Prospects Model”^3^ (SYN 1: October 2018): This “concept paper” was written by the company Synthesis GmbH, which developed the algorithm. It lists information on data, models and parameters as well as some performance measures. One of the specified models used to classify job seekers sparked critical media debates due to potential discriminatory effects (Holl et al., 2018).
3. “Parliamentary Inquiry to the AMS by the Austrian Federal Minister for Labor, Social Affairs, Health and Consumer Protection” (PARLIAMENT: November 2018): Around the same time, the Austrian Parliamentarian Heinisch-Hosek (Social Democratic Party) formulated a parliamentary inquiry to Hartinger-Klein, Minister for Labor, Social Affairs, Health and Consumer Protection. This document comprises the questions and answers of the parliamentary inquiry that is mainly directed at the organizational process the AMS algorithm is supposed to be embedded in Heinisch-Hosek (2018).
4. “Labor Market Policy Targets” (MINISTRY: February 2019): This policy document written by Hartinger-Klein, Federal Minister for Labor, Social Affairs, Health and Consumer Protection summarizes labor market challenges and policies. It gives insights into the larger socio-political context and the demands that the AMS algorithm is supposed to remedy (Holzer, 2019).
5. “Report of the Ombudsman Board to the National Council and the Federal Council 2018: Monitoring of the Public Administration” 2018 (OMB BOARD: March 2019): In the aftermath of the previously mentioned controversies the Ombudsman Board was asked to evaluate the algorithm. This report is the result of this request (Volksanwaltschaft Österreich, 2019).
6. “Inquiry to the AMS by the Ombud for Equal Treatment” (EQUAL BOARD: April 2019): In early 2019, further public oversight bodies and non-governmental organizations started to officially investigate the algorithm. The first one was the “Ombud for Equal Treatment,” which formulated 20 comprehensive questions dealing with the data used for the algorithm, models and parameters of the algorithm, the expected role of the algorithm in the daily practice of the AMS, potential consequences to be expected, as well as planned modes of evaluation. The answer was signed by the president of the AMS (Buchinger, 2019).
7. “Personal Probability Statements (‘Algorithms')” (SYN 2: May 2019): This “Companion Volume to the Documentation of the AMS-Employment Prospects Model” is the second paper published by Synthesis GmbH and focuses on aspects of social compatibility of the algorithm. It may be seen as a response to the critical public and policy debates the test phase of the algorithm sparked (Holl et al., 2019).
8. “Inquiry to the AMS by Epicenter Works” (EPI WORKS: August 2019): The latest inquiry for the time being was formulated by the NGO Epicenter Works concerned with and lobbying for digital rights. The document contains eight specific questions dealing with technical details, data, models and parameters, expected role of the algorithm in the AMS practice, audit possibilities, as well as costs of the algorithm and its maintenance in the future. Additionally, it comprises further technical details of one of the models used for the algorithm. The answer was signed by the head of the AMS president's office (Wilk, 2019).

To analyze both content and context of these heterogeneous documents we follow a constructionist research tradition that is “concerned with the processes through which texts depict “reality” rather than with whether such texts contain true or false statements.” (Silverman, 2015, p. 285). We treat our documents as “social facts” (Coffey and Atkinson, 1997; Atkinson and Coffey, 2011), i.e., we analyze them for “what they are and what they are used to accomplish” (Silverman, 2015 quoting Atkinson and Coffey, 2011, p. 58). Our final selection of materials comprises very different types of documents, hence the context of their production and the timeline of the various documents require consideration. Using a Grounded Theory approach we identify common rhetorical patterns and frames used in our materials (Glaser and Strauss, 1967; Bowen, 2009). Their content is analyzed along categories that result both top-down from our research questions and bottom-up from the empirical material itself. When indicated, we step outside of the “narrative material itself and consider questions, such as who produces particular kinds of stories, […] what are the consequences, and how are they challenged”(Silverman, 2015 quoting Gubrium, 2005, p. 252). In this way, we do not only identify the rhetorical and technical shaping of the algorithm, but also the overarching purposes, agendas and legitimation strategies in the context of larger socio-political trends. This allows for understanding the AMS algorithm in-the-making, but also overarching demands the system is expected to serve. Finally, this approach allows the study of the AMS algorithm despite the fact that its specific operationalization is as of yet unclear, which prohibits other empiric approaches, such as for example, *in-situ* interviews with AMS case workers or job seekers.

## 3. Empirical Analysis: The Socio-Technical Shaping of the AMS Algorithm

The empirical analysis of the socio-technical shaping of the AMS algorithm is presented in three sections. First, to set the stage, we start with reconstructing the technical specificities of the algorithm and its potential biases. We then elaborate central guiding principles in the shaping of the algorithm and its controversial aspects. Finally, we discuss how the algorithm is supposed to be embedded in counseling practices and which challenges this may entail in the future.

### 3.1. Technical Reconstruction of the Algorithm and Its Biases

To understand the inner workings of the AMS algorithm this section discusses the data used for the algorithm and their origin, models and variables used to classify job seekers, as well as potential biases and discrimination that may result from using the algorithm^4^. Any algorithmic system is dependent on data, which is either pre-existent or newly created for the purpose of the algorithm. This also holds true for the AMS algorithm, which is built on data of and about job seekers that subsequently gets fed into a machine learning algorithm.

The exact origins of all data used to craft the variables for the prediction model have not been made public so far. Answering the inquiry by the Ombud for Equal Treatment the AMS president argues that the data originates from the already established data warehouse of the AMS, which mainly contains data from two sources (EQUAL BOARD, question 2). First, some data is collected through self-reporting of job seekers upon registration with the AMS through their online tool^5^ and during other interactions with the AMS. Second, social security data from the Main Association of Austrian Social Security Institutions^6^ is used to complete the job seeker's profile. This organization collects and stores personal data, such as date of birth and gender, as well as data related to the individual employment history through statements of former and current employers.

Crucially, as far as is known, no data is collected specifically for the AMS algorithm. Instead, data gets re-purposed for the profiling systems. The public employment service law^7^ provides the legal foundation for this practice (Arbeitsmarktservicegesetz, 2019; Lopez, 2019) and includes the collection of diverse data about job seekers, including health impairments that could affect employability, as well as obligations of care—both will become relevant in the further discussion of the algorithm. It is important to note that the AMS is also allowed to collect data about employers, including their recruiting and hiring behavior, and data about job openings more generally, but that it has not used this kind of data for the AMS algorithm.

#### 3.1.1. The Regression Models and Their Implications

According to the initial “concept paper” by Synthesis GmbH (SYN 1) the most fundamental component of the AMS algorithm are multivariate logistic regression models. They are used to predict the short- and long-term employment prospects of job seekers in the care of the AMS. These forecasts are the basis for the classification into the three categories A, B and C.

The regression models are built by relating nominally scaled, independent variables based on personal data and information on regional labor markets to two dependent target variables. These model the achievement of job seekers to find gainful, non-subsidized employment of either at least 90 days within a 7-months period or at least 180 days within a 24-months period. In order to calculate the coefficients for each of the nominally scaled independent variables, Maximum-Likelihood estimation is employed. The coefficients delineate the significance of predictor variables to forecasting a target variable. The result of this logistic regression is a value in the range of 0–1. In order to make the calculated scores of the logistic regression applicable to providing classification recommendations, a threshold, also referred to as the cut-off point, has to be chosen that demarcates the groups.

In the following, we refer to regression models derived through one of the target variables as either the short-term (7-months period) or long-term (24-months period) prediction models. They are used to forecast the ex ante employment prospects of current AMS clients, or as referred to in SYN 1, the “prospects of labor market integration value”^8^ (IC). Job seekers are classified into one of three categories based on their IC:

- Group A: High IC score in the short-term prediction model of 66% or above
- Group B: Moderate IC score in both prediction models that is not classified as Group A or C
- Group C: Low IC score in the long-term prediction model of 25% or below.

The assignment of a job seeker to group A is contingent on the short-term prediction models, whereas the assignment to group C is determined by the long-term prediction models. Job seekers not assigned to either group A or group C are sorted into group B. The use of strict IC thresholds for classification is problematic in practice, as a single percentage point might determine whether a job seeker will receive full support of the AMS or not. Synthesis does not provide any guidance on how these edge cases will be handled in practice (SYN 1). Later in the process, after several inquiries have been made to the AMS (OMB BOARD, EQUAL BOARD), Synthesis' second paper describes the algorithm as a “second opinion” rather than an automated decision (SYN 2). Synthesis further states that the time spans in the model of the short-term and long-term target variables were chosen by the AMS, but does not explicate how and why (SYN 1). However, these decisions strongly impact the lives and potential prospects of job seekers (depending on how they were classified), and embody the values and norms inherent to social policies. This ambiguity on how and why certain time spans were chosen for the target variables was also criticized by the Austrian Ombudsman Board^9^ (OMB BOARD).

As stated earlier, thresholds have to be defined to make the logistic regression usable as classification mechanism: only if the predicted likelihood is above or below the threshold, the data point is classified as a specific class, i.e., class A or C. The IC score thresholds for the assignment to groups A and C were chosen by maximizing the sum of the sensitivity (correct prediction of successful integration *ex post*) and specificity (correct prediction of unsuccessful integration *ex post*) of the respective models according to SYN 1. However, the supposedly strict adherence to this approach by the developers is contradicted by a more recent statement (EQUAL BOARD, question 13) saying that the thresholds were purposefully chosen in order for errors and ambiguities to more likely accumulate in group B, which would lead to fewer job seekers receiving less support due to wrong classification.

SYN 1 lists the predictive variables of the AMS algorithm shown in Table 1 (with the values of the variable set “prior occupational career” defined in Table 2). All variables are modeled as binary assignments (“yes”/“no”) where applicable, or as binary assignment to one of the potential options (for instance, “production sector: yes”). Every option is assigned a coefficient in each of the models, and the sum of all coefficients for a given person results (after a logistic transformation) in the IC score for that person.

**Table 1:** All variables that are part of the statistical model.

**Variable**	**Nominal values**
Gender	Male/Female
Age group	0–29/30–49/50+
Citizenship	Austria/EU except Austria/Non-EU
Highest level of education	Grade school/apprenticeship, vocational school/high- or secondary school, university
Health impairment	Yes/No
Obligations of care (only women)	Yes/No
Occupational group	Production sector/service sector
Regional labor market	Five categories for employment prospects in assigned AMS job center
Prior occupational career	Characterization of variable listed in Table 2

**Table 2:** Variable characterizing prior occupational career through four subvariables.

**Subvariable**	**Nominal values**
Days of gainful employment within 4 years	<75%/≥75%
Cases within four 1 year intervals	0 cases/1 case/min. 1 case in 2 intervals/min. 1 case in 3 or 4 intervals
Cases with duration longer than 180 days	0 cases/min. 1 case
Measures claimed	0/min. 1 supportive/min. 1 educational/min. 1 subsidized employment

While some of the assignments for given values are described relatively clearly in SYN 1 (the age group, for instance), others remain entirely vague (e.g., health impairment). In addition to the different predictive variables, the system differentiates between the following four populations based on the completeness of the individual's employment record (EQUAL BOARD):

- Job seekers with a complete historical employment record of 4 years prior to model generation
- Job seekers with an incomplete or “fragmented” employment history^10^
- Job seekers with a “migration background”^11^
- Young adults.

The last three groups are characterized by their composition of job seekers with incomplete or fragmented employment histories and/or data. The classification models were clearly crafted to handle the increased uncertainty due to less data being available, but it has not been made public how and why the specific grouping was chosen.

The prediction system we have described so far is comprised of two IC models for four populations. Since job seekers are imagined to remain in the care of the AMS usually for some time, models to account for the following 12 milestones—depending on the length of the current case—are also part of the system: at the beginning of the case, every 3rd month until the 2-years-mark, as well as at the 30th, 36th, and 48th month. These three dimensions (two target variables, four populations, 12 milestones) result in a total of 96 models.

SYN 1 lists the precision values for both the short-term and long-term prediction models for nine select populations at certain milestones:

- Incomplete data: “migration background” after 6 months; Vienna beginning of the case
- Complete data: beginning of the case, after 12 and 24 months, respectively
- Complete data and beginning of the case: women; men; Salzburg; Carinthia.

Interestingly, the geographic contexts listed (“Vienna,” “Salzburg,” “Carinthia”) do not appear within the models otherwise; the only reference to geographic location could be found in the variable “regional labor market,” but it is unclear why and how the precision of these specific subpopulations was evaluated or how they relate to the models and populations.

The reported precision values vary between 80 and 91% for the year 2018. Up to this date, no information was provided on the precision of other models that were not included in the document being applied to (sub-)populations, calling into question the overall purported precision of at about 81%. For instance, no information is provided for job seekers with “migration background” at the beginning of the case.

#### 3.1.2. Bias and Discrimination

A core issue of the public controversy over the AMS algorithm has been concerns of discrimination, particularly of women, people with disabilities and women with care obligations. Since gender, dis/ability, and other ascribed characteristics feature prominently in the models of the AMS algorithm, investigating these concerns is necessary. The justification for using these sensitive attributes includes the argument that the coefficients generated for these variables represent the “harsh reality” of structural discrimination on the job market. Hence, it would not be discriminatory to use this information the way it is done (OMB BOARD, EQUAL BOARD); an argument we challenge in several ways.

Preexisting inequalities appear to be inscribed into the AMS algorithm through the data it is based upon and the way they get processed. Specifically, the algorithm incorporates the cumulative disadvantage (Gandy, 2016) of currently marginalized groups on the labor market represented in the algorithm through variables, such as gender. Due to this mechanism, previously discriminated or marginalized groups are more likely to be classified as part of group C, which in turn reinforces existing inequalities as more discriminated populations of job seekers are more likely to receive less support. Additionally, the AMS algorithm reifies the category of ‘long-term unemployed job seekers' (group C). At this point it is difficult to assess the dynamic this entails, but, as Porter (1996) argues, “measures succeed by giving direction to the very activities that are being measured.” (p. 45) Put differently: classifying job seekers as ‘hopeless' can trigger a process in which resource deprivation can lead to the realization and validation of the prediction.

These negative impacts on employment prospects are fed back into the algorithm continuously as it is updated with newly collected data. This feedback loop might, over time, further decrease employment prospects of marginalized groups. In practice, however, the described effects of this profiling system are more varied and nuanced, as case workers may deviate from algorithmic recommendations (see section 3.4). Furthermore, other policies factor into these effects as well: certain groups may still receive full support under a policy mandating the equal funding of men and women, which may remedy some of the detrimental effects of this feedback loop. However, although the AMS initially argued that policies like these protect, for instance, women from discrimination by the algorithm, the policy in question was recently discontinued (Lopez, 2019).

In addition to fueling the cumulative disadvantage of marginalized groups directly, other variables contribute to this effect indirectly. We illustrate this with the variable “regional labor market,” which models the situation of the labor market in any given AMS office's region based upon the calculated ratio of “new job seekers registered” to “job seekers taking up employment,” i.e., *incoming* vs. *outgoing* job seekers. In practice, this carries the danger of becoming a proxy variable for historic socioeconomic disadvantages predominant in an AMS office's region, implicitly representing a marginalized group associated with cumulative disadvantage, for instance, minority ethnic groups (Gandy, 2016). Another way inequalities on the labor market get introduced implicitly into the algorithm is the separation of populations with incomplete data into three subpopulations: job seekers with an incomplete or “fragmented” employment history, “migration background” and young adults. These groups are particularly vulnerable as incomplete employment histories can represent a disadvantage on the labor market and therefore presumably lead to higher probability of an assignment to category C (Lopez, 2019). The possible exception are “young adults' under the age of 25,” who continue to receive full support no matter what group assignment (EQUAL BOARD).

To this date, no full explanation has been provided as to why the population of job seekers with incomplete employment histories was split up into three. In particular, the population classified as having a “migration background” (which is itself a problematic term, see e.g., Will, 2019) represents a marginalized group on the Austrian labor market, making them prone to the same effects of cumulative disadvantage. A reduction of spending on this population would also align with intentions voiced by the former Austrian government, which planned to cut AMS support measures for refugees (MINISTRY). This is further underlined by the high proportion of refugees assigned to group C as stated in EQUAL BOARD (question 7). The AMS president claims that—since “ethnic descent”^12^ is not taken into consideration in the predictive system—anti-discrimination laws are not violated (EQUAL BOARD). But there is no documentation on how the AMS defines “migration background” in this context or how group classification is conducted for this population. Ultimately, it is very likely that “migration background” is to some degree correlated with “ethnic descent.”

Although such modeling practices may improve the accuracy of the whole system, they may also entail disparate impacts (Barocas and Selbst, 2016), as incomplete data records may result in higher error rates. Performance metrics may vary based on the evaluation of the performance of the system for given sub-populations. This problem is commonly referred to as the Simpson's paradox (Bickel et al., 1975): on some level of population granularity, performance measures, such as precision or accuracy are equal across sub-populations, while at a different level, the performance measure varies. There have been numerous studies on how to identify, and ensure, “fair” algorithms in the sense that the algorithms perform equally well across sub-populations (see e.g., Friedler et al., 2016; Wattenberg et al., 2016; Gajane and Pechenizkiy, 2017). But without access to more detailed data, it is impossible to know if there are stronger variations across sub-populations in this case. SYN 1 lists different precision values for classifications into class C from 81 to 86%, depending on the province;^13^ similarly, precision values are slightly different for men and women. Given these observations, already marginalized sub-populations might be adversely affected by more frequent miscategorizations of the system.

Issues of cumulative disadvantage, feedback loops and disparate impacts lead to biases and indicate various types of discriminatory mechanisms inscribed in the system. First, biases are introduced into the algorithm through the use of coarse variables as predictors. For example, disadvantage and discrimination are not affecting all job seekers that are part of certain marginalized groups the same way: for instance, in some sectors, women are not disadvantaged, but even preferred over men. Conceptualizing “women” as a homogeneous group, is problematic. In a similar manner, employment prospects may vary highly among individuals employed in the service and production sectors, respectively. In the case of the variable “occupational group,” this issue is particularly apparent: as stated in SYN 1, this variable is modeled as a binary choice between the *production* and *service* sectors, and reference an AMS internal occupational taxonomy of nine groups. This classification is most likely based on the International Standard Classification of Occupation of ISCO-08 as maintained by the International Labor Organization (ILO^14^.) that is mentioned at the AMS's own occupational taxonomy website^15^. Thus, the production sector would include no <6 separate areas^16^, whereas the service sector includes an additional four^17^. The ninth major group in the taxonomy, Elementary Occupations, is notably absent from the list presented in SYN 1, and the other major groups cast serious doubts at the sectional grouping under the labels “production” and “service.” Frequent overlaps between tasks and industries make a binary assignment of one or the other label for many job seekers a highly questionable practice, particularly given that the ILO refrains from making such a grouping themselves. Given this apparent heterogeneity, it is highly likely that the system is biased significantly for many job seekers, depending on which occupational group they get assigned to based on their actual sectors. Second, biases are introduced through blurred, contingent and dynamic boundaries of the categories of the AMS algorithm. For instance, some AMS locations are geographically closely related (e.g., districts of Vienna), but are otherwise heterogeneous in terms of demographics of job seekers. Consequently, when searching for employment, job seekers may not be limited to the regional labor market of a specific AMS location, but their IC scores are still influenced by the location's calculated prospects. It stands to reason—for instance due to the interrelatedness of regional labor markets—that the measure may be influenced by many more factors than simply the favorability of the local job market. Another issue arises if a region is strongly dominated by one industry, or even a single company. Any developments that affect this industry (e.g., mass layoffs) may also affect the IC scores and access to resources of job seekers that are not part of this industry.

### 3.2. Central Guiding Principles and Controversial Aspects

In this subsection, we take a closer look at the main principles that explicitly or implicitly contributed to the shaping of the AMS algorithm. While the various analyzed documents explicitly refer to goals of efficiency and effectivity as guiding principles of the system, the implicit framing of unemployment that comes along with them affects the system specification as well.

#### 3.2.1. Efficiency and Effectivity

The AMS algorithm is a “project to increase efficiency,” emphasizes the Court of Auditors (Rechnungshof, 2017, p. 82). Raising the efficiency of the AMS has become a pressing concern due to rising numbers of job seekers, a stagnating budget and limited human resources (COURT OF AUD). Consequently, the Ministry of Labor, Social Affairs, Health and Consumer Protection rhetorically shapes the AMS algorithm as a “key to efficient labor market management” (MINISTRY, p. 3). It further argues that the algorithm should “provide support that allows the AMS to offer good counseling even with reduced consumption of resources” (MINISTRY, p. 5). More specifically, the AMS algorithm is expected to realize “potential savings regarding personnel and resources” (MINISTRY, p. 3). Fiscal austerity occurs as one of the main drivers for the AMS algorithm. Both the Court of Auditors and the Government (COURT OF AUD, MINISTRY) pressured the AMS to increase its efficiency in times of economic downturn^18^. While the initial goal was to support an increasing number of job seekers with a stagnating budget, the more recent expectations voiced by the ministry, frame the AMS algorithm as a means to enable further budget cuts (MINISTRY).

An equally important objective of the system is to optimize “investments” in job seekers based on their predicted short- and long-term unemployment. The algorithm provides the AMS the ability to render job seekers “objectively calculable” (Rose, 1998, p. 90) and accordingly, they are supposed to get access to resources in a way that maximizes the effective use of resources: “Particular attention must be put on increasing the training effectiveness, so that only training is utilized that is actually suitable for an integration into the labor market”^19^ (MINISTRY, p. 4). The AMS algorithm is a means to sort job seekers to maximize their “organizational utility” (Rose, 1998, p. 90)—which means to the AMS, to maximize the number of job seekers that successfully, and sustainably get re-employed (Penz et al., 2017). Following this logic, job seekers who are deemed unlikely to find new employment are a waste of resources. According to the AMS president, these costumers should get access to offerings that are claimed to be a better fit for their situation than traditional trainings in the form of up-skilling (EQUAL BOARD). More importantly, however, these offerings are designed to require less resources (COURT OF AUD).

#### 3.2.2. Individualization of Unemployment

In line with the activation paradigm, the AMS algorithm has a strong tendency to individualize the causes of unemployment. Broadly speaking, there are two parts of each model that influence a job seeker's IC score: firstly, the variables and the values they assume, and secondly, the variables' coefficients. In combination, they produce a job seeker's IC score. In this section, we take a look at both parts and analyze to what extent they attribute agency to the job seekers and other actors.

The vast majority of variables are based on official records of the job seeker, e.g., gender, health status, education, etc., and are tightly associated with the individual. Only the variable “regional labor market” is not solely related to the job seeker but also to contextual circumstances (at least to some extent). The assignment of an AMS location and thus the variable is still based on the job seekers home address, and not their choice of location where they seek employment. In contrast to this, the variables' coefficients are highly dependent on the wider economy. Since they stem from a statistical process involving the data of hundreds of thousands of people, it is much more appropriate to locate the reasons for certain (dis-)advantages of populations in the labor market within the hiring practices of companies and other developments beyond the job seeker's control, than with the actions or choices of the individual.

Most of the variables are difficult or impossible to influence by the job seeker. Age cannot be changed, for example, but can have considerable impact on the IC. The choice of the three age brackets utilized within the system seem particularly prone to produce problematic edge cases: given the strict thresholds, a single year of age can make the difference between gaining access to a training measure or being refused such support. Other ascriptions, such as health impairment and citizenship, are highly contingent on established standards, norms and practices, both within the AMS and broader society. As an example, a third gender has been officially recognized in Austria since a supreme court ruling in 2018, but is not represented in the current models. Other variables could only be influenced preemptively by the job seeker by making considerable sacrifices, such as choosing not to have children.

Some of the variables are based on past events, such as previous contacts with the public employment service and the number of days in employment. Neither can be actively changed in the present, although over time, some of these past events may no longer be taken into account because they occurred too far in the past. The number of measures the job seeker has participated in straddles a middle ground: While oriented toward the past, this is actually something the job seeker, if supported by the AMS, has some (limited) agency over. Given that the job seeker's classification gets re-calculated several times a year, this provides an opportunity to actively affect the job seeker's classification.

The variables' coefficients may change any time the models are recalculated. It is possible, for example, that legal changes (e.g., to stimulate the job market), or economic trends impact the coefficients. Contrary to the variables, however, the coefficients are less visible, partly due to their numeric character—whereas the variables associated with the job seeker are very telling and explicit. In combination, this can create the impression as if it is the job seeker's attributes that influence the IC score due to the variables' high visibility.

Also it is questionable how likely it is that the classification for clients in group C may change by participating in so-called supportive programs. While the AMS' aim is to prepare long-term unemployed individuals to make them “ready” for job search (MINISTRY), any such action and support has only limited effect on their algorithmic classification (likely only affecting the variable “measures claimed” as detailed in Table 2). Skepticism in this direction is also voiced by the Ombudsman Board:

“If the evaluation so far indeed showed that predictions for low prospect in particular are correct, then this additionally means that the AMS didn't react appropriately to this “bad risk” of a person: The support by the AMS didn't improve the prospects of this group of people according to the provided model.”^20^ (OMB BOARD, p. 102).

This concern points to a tension: On the one hand, the AMS shall help in making the job seekers part of group C “job-ready.” On the other hand, if the AMS was successful in supporting the job seeker, this would decrease the correctness of the AMS algorithm's predictions. In order to legitimize the algorithmic classification by ensuring its precision in the future, this could fuel a self-fulfilling prophecy by affecting the practices of the AMS in subtle ways. Adding to the impression of extremely limited agency of the job seekers in group C are plans to “stabilize their personal situation and strengthen their motivation”^21^ (MINISTRY, p. 15). This narrative implicitly paints a picture of long-term unemployed people as unstable and unmotivated. The practice of predicting long-term unemployment, the rhetoric of ‘stabilization' and their reduced access to active labor market programs have the potential to further stigmatize people classified as C. This is particularly troubling, because it can even happen on the first day of unemployment due to the predictive nature of the AMS algorithm: being classified as part of group C does say, after all, that a new job seeker is seen as unlikely to find a stable job within 2 years. In the medium term, this rhetoric can be used to further deprive stigmatized groups of their social rights.

The individualization that takes place with the algorithmic classification can be directly linked to the transition toward a “workfare” state as discussed in the introduction. At the same time, (predicted) long-term unemployed can be seen as not fitting a “workfare” state, as being “hopeless,” or “lost” to this form of society. Only within this logic can minimizing the spending for these job seekers be justified.

### 3.3. Quality of the AMS Algorithm

The AMS algorithm is repeatedly framed as objective and precise. Both claims are used to highlight the system's quality, and to some extent, superiority to practices it shall replace (or augment).

#### 3.3.1. Objectivity

One key argument in support of the AMS algorithm is its proclaimed objectivity: answering the inquiry by the Ombud for Equal Treatment the AMS aims for “the highest objectivity possible in the sense that the predicted integration likelihood …follows the real chances on the labor market as far as possible”^22^ (EQUAL BOARD, question 20).

Source to this claim is the data that enables the AMS algorithm. According to the AMS president in EQUAL BOARD, their data allows the AMS to explore manifold attributes of job seekers and correctly identify those that correlate with the prospect on the labor market. This reference to the amount and variety of data draws on a standard trope of big data, namely that it is a detailed, encompassing and “truthful” representation of “reality,” following the ideal of mechanistic objectivity (Daston and Galison, 2007; Rieder and Simon, 2016; McQuillan, 2018). As scholars in the field of critical data studies and STS have shown, these claims are misleading (Boyd and Crawford, 2012), because they are always produced within a specific context and with a particular goal in mind (Bowker, 2005). Consequently, contrary to the claim of providing a truthful, objective representation of reality, big data can only provide oligoptic “views from certain vantage points” (Kitchin, 2014, p. 133). These objections to the objectivity claim of big data also hold true for the data used for the AMS algorithm.

Datafication and big data have renewed the interest in the idea of “evidence-based decision-making” by policy makers and public agencies (Rieder and Simon, 2016). Basing decisions on quantified “evidence,” embodied by big data, promises to get rid of potentially dangerous subjective human intervention (Rieder and Simon, 2016), to make “objective” decisions “according to nature and not according to prejudice” (Rose, 1998, p. 90). One repeated claim defending the AMS algorithm's superiority is, then, that the case worker's “assessments [of job seekers] turned out rather more negative than calculated by the software”^23^ (OMB BOARD, p. 100). An alternative reason for the judgment of the case worker erring on the side of anticipating vulnerability of long term unemployment could be quite simply a more cautious stance that does not neatly align with the quantitative risk assessment of the AMS algorithm (Sarewitz et al., 2003). The provided explanation implies, first, that the case workers *judged by the algorithm*, seem prejudiced, and second, that the AMS algorithm provides a means to overcome prejudice.

Despite the claim that the model of the AMS algorithm is a faithful representation of “reality,” the AMS does acknowledge that important factors that can contribute to a job seeker's success are missing from the model. Explicitly mentioned is, for example, that soft skills of job seekers could not be modeled, as these are “empirically hard to capture”^24^ (OMB BOARD, p. 100). At the same time, the AMS seems to have had no trouble capturing the complex and manifold dimensions of human health and their impact on *all* potential jobs in a single, binary variable—“health impairment” (see Mills and Whittaker, 2019). Similarly, despite the claimed problems of “biased” case workers, it is ultimately their responsibility to decide the job seeker's group assignment (Volksanwaltschaft Österreich, 2019), which has crucial implications in practice, as we further discuss below.

#### 3.3.2. Precision

This section takes a closer look at performance measures of the AMS algorithm. As detailed above, during the design of the algorithm, performance measures guided the selection of variables and cut-off points (EQUAL BOARD). In computer science and statistics *technical definitions* of performance measures, such as accuracy and precision are not consistent. In the context of algorithmic classification, *accuracy* is a statistical measure that indicates the fraction of individuals out of the full set that got correctly predicted. Closely related to accuracy is *precision*. Precision looks only at one particular group. For the AMS algorithm, precision is the percentage of individuals predicted as, e.g., group A that actually belongs to this group. The complementary measure to accuracy is the *error rate*, the fraction of incorrectly predicted individuals. In the case of binary classifications, two types of errors can be distinguished. An individual can be predicted as group A but belongs to one of the other groups—called *false positive*. Individuals that are predicted as B or C but belong to group A are *false negatives*. In the case of the AMS algorithm this distinction is important because how an individual is misclassified implies access to or denial of particular resources. This distinction between types of errors is lost if only information on the accuracy is provided—precision only shows the fraction of false positives, but contains no information about false negatives.

**Figure 1:**
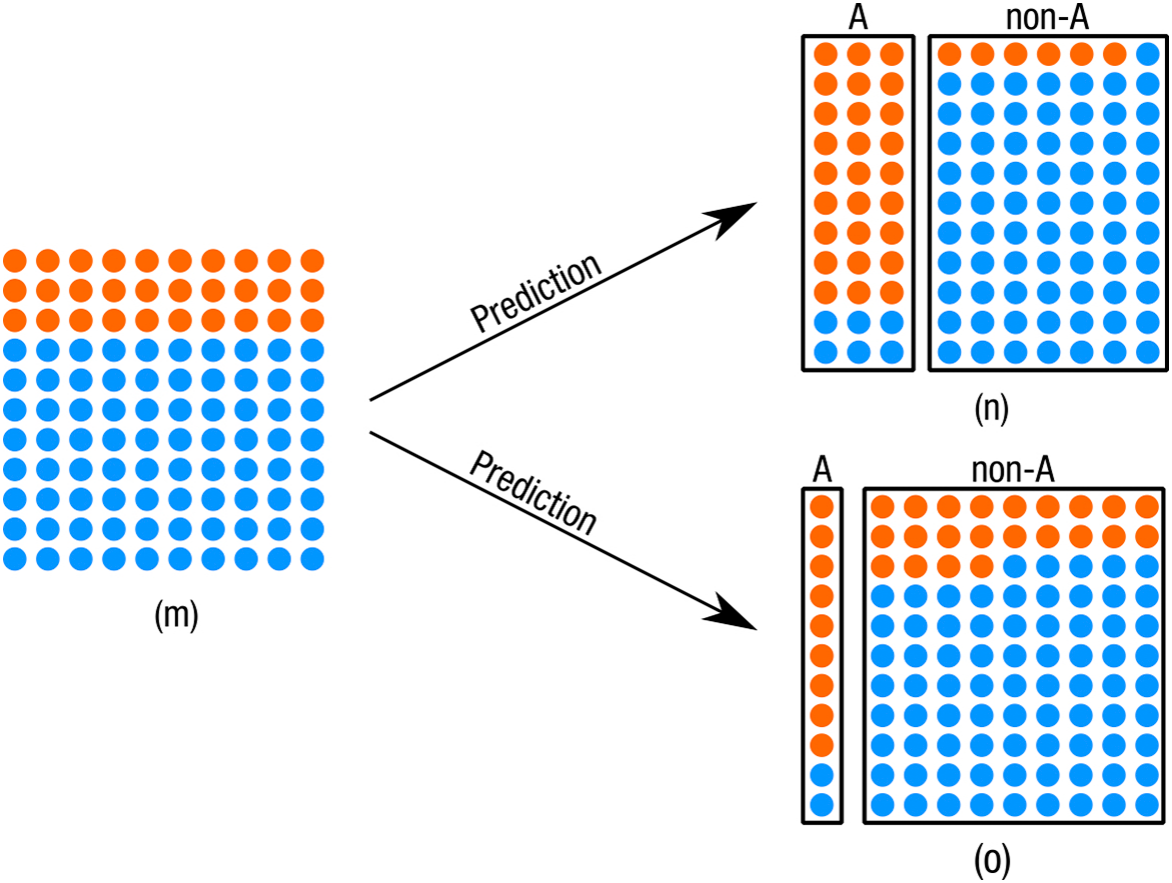
By varying the precision of the predicted group non-A one can vary the size of the group A and retain the same precision for group A. Orange dots are in group A (30%), blue dots are not (70%). The two predictions (n) and (o) both have precision of 80 % for group A, yet different accuracy [88% for (n) and 76% for (o)], and errors.

The first paper by Synthesis GmbH (SYN 1) provides only information about precision. The numbers show precision in the range of 80–84% for group A and 81–91% for group C. The exact values depend on the model (e.g., individuals with “fragmented” employment history) and sub-population (the values are different for women and men and vary by province according to the documentation). No numbers are provided for group B. Providing only precision limits what conclusions can be drawn about the correctness of the predictions. As Figure 1 illustrates, one can vary the size of, e.g., the predicted group A and retain the same precision. What changes are the overall accuracy, the precision of the other class and the number of errors and their type. Consequently, by experimenting with the size of the predicted class A, one can more easily reach desired precision. At this point we have to recall how the thresholds of the logistic regression were chosen. As we have described in section 3.1 there was considerable tinkering and tuning going on, guided by the preferences of the AMS: Robust precision should be maintained for the groups A and C, and classification errors should accumulate primarily in group B (EQUAL BOARD, question 13). This highlights two things. First, the focus on precision as metric (compared to, for example, accuracy or a set of metrics) opens up certain ways of tinkering (and closes others). Second, how certain performance numbers are achieved are not merely “technical,” or “neutral” decisions but laden with value judgements or tacit routines that have to balance a range of trade-offs, something also acknowledged by the Ombudsman Board (OMB BOARD).

Performance measures, such as precision and accuracy are problematic in additional ways. First, this kind of quantitative performance measure is frequently used as a *signifier of quality*. This narrative is taken up by the Ombudsman Board when they state that, even though soft skills are not part of the models, “continuously [conducted] *ex-post* evaluations have shown that the precision of individual predictions would vary in the range of 80–85%”^25^ (OMB BOARD, p. 100). While this passage does not explicitly state that this shows the quality of the AMS algorithm, it is brought forward to counter doubts about the expressiveness of the model. However, it is entirely unclear if the provided performance numbers should be deemed as “good” or not, and on what grounds. Second, using precision (or, more frequently, accuracy) as quality measure is further solidified because it allows quantifiable comparisons across socio-technical systems (Desrosières, 2010). A striking example is a recent OECD report comparing statistical profiling systems used by public employment agencies of OECD member states (Desiere et al., 2019). While the profiling systems vary to a great extent, performance measures provide a neat, and intuitive way to claim that some are ‘better' than others. Note however, that this report shows accuracy numbers for the Austrian system—it is, however, unclear whether this refers to accuracy as defined in this section or whether it actually reports the precision. Consequently, terminological and translation issues may contribute to the inappropriateness of comparing the quality of different systems by focusing on performance metrics. Additionally, quantitative metrics privilege socio-technical systems that allow for quantification—and tend to ignore “aspects of society that resist quantification” (Green, 2018, section 2.1, para.1). The inability of the AMS to capture soft skills and motivation in a quantifiable way makes this point salient.

Both usages of performance metrics as signifiers of quality build upon the assumption that they are “objective” measures that “naturally” come about. As we have shown, this is not the case. Additionally, they reinforce the imagination that algorithmic classification is purely mathematical, scientific, technical, a-political and a means to get rid of value judgements. To draw on a core concept from STS, they are inscriptions produced by black-boxed mechanisms providing “the focus of discussion about the properties of the substance” (Latour and Woolgar, 1986, p. 51)—in this case they provide the focus of the discussion about the new system's quality. Even the AMS itself acknowledges that convincing precision numbers are not the be-all and end-all proof of quality, as the following section will show.

### 3.4. Presumed Integration Into AMS Practices

Since the algorithm is currently in its test phase the full integration of the system into AMS practices is speculative at this point. The usage of the algorithm in practice, however, is envisioned in the materials surveyed in various, partly contradictory, ways. For instance, the initial concept paper (SYN 1) does not mention the operationalization of the system and its integration into the daily work practices of the AMS at all. Only after the AMS was challenged to share their ideas about the actual integration of the algorithm into counseling practices (inquiries by EQUAL BOARD & EPI WORKS), did Synthesis publish its second paper on the “social compatibility” of the algorithm (SYN 2). This indicates that many of the complex questions about the day-to-day usage of the algorithm in practice were not considered preemptively before rolling out the test system.

At the core of multiple imaginaries about the social embedding of the system is the complex relationship between the algorithm and the case worker. While the “objectivity” of the algorithm is praised in several ways, the human is put back into the loop when speaking about the actual counseling practices, enacting notions of the case worker as a “social corrective.” As stated in OMB BOARD: “The algorithm in use is only expected to be a measure of ‘technical support' for the AMS workers; the decision as part of the support process, in the end, will have to be made by the workers themselves”^26^ (OMB BOARD, p. 99). Accordingly, the AMS algorithm is framed as a “support system” rather than a fully automated decision-making system. This aspect is relevant for several reasons: First, the responsibility for the final decision is delegated to the case worker. In EQUAL BOARD, the AMS states that the case workers would know that the “Personalized Labor Market Assistance System” is an “assistance system, which does not take over their responsibility, but that helps them to fulfill their responsibility”^27^ (EQUAL BOARD, question 14). Second, SYN2 explicitly frames the algorithmic suggestion as a “second opinion,” which helps to legitimize the introduction of the algorithm despite its apparent, and acknowledged, weaknesses. Even the AMS president speaks of “weaknesses of the system,” which should be “corrected” (EQUAL BOARD, question 14) by the individual case worker. Similarly, the case worker is expected to consider job seekers' soft skills and any other attributes which were not integrated in the algorithm, such as motivation, engagement or their social network. In all these examples, the case worker has to make up for the system's shortcomings, either in a corrective way or by assuming responsibility for a decision. Thus, the rhetorical pattern of the “social corrective” helps to stabilize the algorithm within social practices by framing it as a mere add-on rather than a revolutionary technology.

This, however, is contrasted by high expectations, specifically in terms of increased efficiency and effectiveness, the overarching guiding principle of the algorithm. Reaching these goals, however, would require a change of practice to make both the expected budget cuts and the envisioned improvements in the counseling process feasible. The tension between the notion of the case worker as a “social corrective” and the notion of the algorithm as calculating an “objective truth” hence poses crucial challenges for the introduction of the algorithm into AMS practices. In particular, case workers may be confronted with contradictory demands. First of all, case workers may find themselves torn between following algorithmic suggestions to speed up the counseling process, and communicating, discussing, correcting and “overruling” the algorithmic system in dialog with job seekers (EQUAL BOARD). While the algorithm is imagined as contributing to more efficient counseling processes, it seems to rather create new duties for case workers. According to the AMS president, case workers are expected to communicate the suggested classification to the clients (EQUAL BOARD), who should be informed about the most influential factors which led to the assignment. Additionally, the case workers' reasoning for overruling a classification decision should be imparted (EQUAL BOARD, SYN 2). To fulfill all these requirements, however, case workers would need to take even more time for the counseling process. Subsequently, significant concerns regarding time constraints being possibly detrimental to the quality of the case worker assessment were voiced in the parliamentary inquiry (PARLIAMENT). This constitutes an alarming development, since the counseling periods are comparatively short in Austria, as case workers are confronted with heavy work loads already (Penz et al., 2017). Accordingly, even Synthesis warns against letting the algorithm be turned into the “first opinion” (SYN 2).

Furthermore, case workers need to find a balance between algorithmic suggestions and their own instincts, which might not always be in line since they follow different logics and rationales. While the algorithm has a mathematical conception of risk based on historical data, the case workers calculate employment prospects in completely different ways based on experiences and social skills (Sarewitz et al., 2003). This raises questions of the comparability of the different types of assessments and how to integrate them in practice. Finally, case workers may be confronted with algorithmic biases and inscribed inequalities that can implicitly reinforce and strengthen their own prejudices or create new ones. In the worst case, this could create a feedback loop in which case workers see their prejudices confirmed by the algorithm and, in turn, treat certain groups of people differently. Consequently, the situation of disadvantaged groups may worsen, which is fed back into the algorithm again. A study on similar profiling system used in Poland (Niklas et al., 2015), shows that counselors felt an increased suspicion toward job seekers who asked how certain decisions were calculated by the system. This indicates that even new forms of stigmatization may emerge with the introduction of such a system.

All the challenges and tensions that may arise when introducing the algorithm in the actual AMS practices call for special training of case workers, in particular awareness/anti-discrimination training. The AMS further suggests technical education that would help case workers assess the algorithmic suggestions they are confronted with as well as their precision and error rates (EQUAL BOARD). This, however, is tricky since case workers have no way of knowing when the predictions of the algorithm are wrong. As the categories used in the modeling of the variables are quite coarse, even information on which factors lead to a certain classification are very limited. Furthermore, it is difficult to relate the individual situation of a job seeker to the algorithmic classification that is based on patterns in collective historical data. Consequently, the opacity of the algorithmic predictions may severely limit the agency of case workers. The danger that case workers are demoted to “rubber-stamping” algorithmic decision-making is indicated by experiences with the Polish profiling system that showed that case workers hardly ever adapted classifications suggested by the system Niklas et al. (2015).

The framing of the algorithm as a “second opinion” also has legal implications. First, it allows the AMS to circumvent “limitations and safeguards” put in place through the GDPR since they only apply to fully automated decision-making systems (Wagner, 2019). Second, it enables the AMS to escape more specific legislation too: the AMS is subject to the Equal Treatment Act, which prohibits unjust unequal treatment based on gender, parenthood, ethnic background, and several other aspects. Including the attributes in the system is legally only possible due to the fact that the algorithm is framed as a mere support system and the final decision is delegated to case workers (Lopez, 2019, p.13); even though case workers may practically be forced to follow algorithmic suggestions due to both the intransparency of the algorithm and the limited time they have for the counseling practice.

## 4. Discussion

The previous analysis has shown that the AMS algorithm should not be seen as a mere technical tool, but rather as a socio-technical endeavor both embedding and reinforcing certain socio-political goals. In the first part of our analysis, we have shown how the shaping of the algorithm is influenced by technical affordances, but also by social values, norms and goals, such as using historic personal data to calculate future job prospects. Moreover, we have discussed potential forms of bias and discrimination due to the fact that past inequalities are encoded in the classification models to sort job seekers into the three categories with different support measures attributed to them. Secondly, we have discussed central guiding principles of the shaping of the algorithm and how they partly contradict each other. While it may sound like a win-win situation to develop an algorithm that is supposed to raise both efficiency and the quality of the counseling practice, our analysis has shown that certain tensions arise when looking at the multiple socio-political expectations the algorithm is supposed to fulfill. In the context of the efficiency narrative, the algorithm is framed as a tool to speed-up the counseling process due to its “precise” classification system and hence evidence-based decision-making abilities. In discourses on the embedding of the algorithm in social practices, however, the algorithm is described as a mere “second opinion” whose weaknesses need to be corrected by the human case worker. This tension between the “objective algorithm” and the case worker as a “social corrective” responsible for the final decision entails several implications for the counseling practices, as we have discussed in detail. It further enables the AMS algorithm to escape several legal frameworks, such as the GDPR or the Equal Treatment Act that only apply to fully automated decision-making systems.

Moreover, questions of accountability are raised in different ways if the algorithm is framed as an automated decision-making system or a mere support system. The increased interest in algorithmic decision-making in recent years has given rise to numerous calls for rules and guidelines for the ethical design and implementation of such systems. What unites many of these guidelines [e.g., Pinto, 2017; US Public Policy Council (USACM), 2017; Larus et al., 2018] is the argument that, in order to utilize them in a safe, fair and ethical manner, the accountability of these systems must be guaranteed. While transparency as a guiding principle is not the only factor—and certainly not a determinant one (Ananny and Crawford, 2016)—the apparent opacity of many algorithmic systems represents a serious hindrance to holding them or their creators accountable (Pasquale, 2015).

In the case of the AMS algorithm, questions of transparency are interesting in and of itself, as we now briefly discuss before raising broader conclusions. On the one hand, transparency as guiding principle is mentioned numerous times in media reports and interviews; even the Austrian Ombudsman Board praises the efforts by the AMS of trying to be transparent (OMB BOARD). On the other hand, there are a number of issues that tarnish this well-presented image of transparency. First, the format of the methodological documentation, as presented in SYN 1, seems designed to remind the reader of a technical specification, with sometimes overly detailed descriptions of minute details, while at the same time omitting large and extremely relevant information. For instance, a detailed description of logistic transformations beyond simply answering why it is being used in this case is provided, but many variables and concepts remain woefully under-specified (for instance, “health impairment” or “obligations of care”). Second, the document fails to explicate the exact nature of the data collection, cleaning and stewardship practices; only through various inquiries, such as the one by the EQUAL BOARD, some more relevant data sources came to light. Third, the number of models, model coefficients, and the complete list of error rates/precision for the vast majority of models were omitted in the document, seriously calling into question the proclaimed goal of transparency.

Finally, even the (presumably) complete list of variables often raises more questions than it answers, for instance how the extremely simplified measure of incoming vs. outgoing job seekers could reasonably represent the regional labor market. Specific modeling choices (such as the grouping via “migration background”) that seem value-laden, purposeful and significant are not sufficiently explained within the document. Rather, most questions about the reasoning behind these choices are answered with supposedly technical necessities (EQUAL BOARD).

The question of transparency is closely linked to the question of accountability, which is a complex issue in general. In the case of the AMS algorithm, *ex-post* accountability could mean the possibility to demand an explanation and justification for the classification after a job-seeker has been classified. There are no formalized processes for this described in the documents SYN 1, SYN 2, or the EQUAL BOARD. Additionally, the very nature of the system and the processes involved in reaching a decision are obviously difficult to justify for any single case. The only possible explanation that could be given to the job seeker—i.e., the specific assignment of coefficient to their personal attributes—cannot explain the multitude of factors that contributed to the forming of those coefficients. Furthermore, it is unlikely that the existing technical language and documentation would be sufficient to explain the necessary details to anyone without prior education in the fields of statistics, machine learning or computer science. Thus, in terms of *ex-post* explainability and, subsequently, accountability, the speculative operationalization of the algorithm is woefully lacking and provides little evidence of supporting a critical inquiry by the general public or affected individuals.

## 5. Conclusion

In the previous sections we have discussed the tensions and ambivalences that the socio-technical design of the AMS algorithm-in-the-making entails. Based on this, our conclusion traces the way the algorithm reflects value-laden judgements of system designers, entrepreneurial practices of the AMS and the “logic of workfare” (Penz et al., 2017). Despite the crucial implications that the introduction of the AMS algorithm may pose in practice, it necessarily relates to how unemployment has been discursively framed and handled in day-to-day business prior to the system. It needs to be seen as a continuation of previous practices of the AMS and in light of the neoliberal shift in Austria's social security system indicating a transformation of the welfare state toward a workfare state with its activation paradigm, as argued in the introduction. Our analytical framework reveals at least three closely interrelated levels of how activation policies and the modeling of data collections on unemployed citizens are coproduced and how they become discursively and materially manifest in and through the algorithm:

First, our analysis traces the co-construction of the algorithm and previous and emerging entrepreneurial practices of the AMS. Pollitt and Bouckaert (2011, p. 10) point to a number of general practices that characterize the new managerialism in the field of unemployment. In order to save money and to increase efficiency, administrative units or even individual employees in the public sector compete with each other based on the measurement of their performance. The most important performance indicator records the fast and sustainable reintegration of job seekers into the labor market (Penz et al., 2017). This is reflected in the short-term and long-term prediction models of the AMS algorithm and the corresponding profiling of job seekers into the groups A and C. In the name of efficiency, the purpose of the algorithm is to speed-up the counseling process and to support evidence-based decision-making at the same time. To advocate the usefulness and quality of the system, the AMS praises the objectivity of the algorithm and uses technical performance metrics, such as precision of the prediction to support their argument. The claimed neutrality, objectivity and precision of the algorithm fuels the idea of the efficiency of bureaucratic work and ultimately of a “lean state.” However, as our analysis shows, the precision of the algorithmic prediction cannot be taken for granted in practice and case workers are supposed to act as a “social corrective.” In addition to the burden of justifying any changes of the “objective” classification of customers resting with the case worker, the responsibility to correct the system and to make cost efficient decisions is ultimately delegated back to the case workers as well. “[T]he public workforce consists of ‘activated activators,”' as Penz et al. (2017, p. 557) conclude in their study of AMS practices prior to the introduction of the algorithm. This leaves case workers “torn between standardized efficiency and customer-orientation” (Penz et al., 2017, p.556). Ultimately, case workers either rely on the precision of the system or prioritize their own experience and consultations with customers^28^. The coproduction of efficient counseling practices and objectified (semi-)automated decision-making re-enacts this tension inherent to the activation paradigm.

Second, our analysis hints to the co-constitution of citizens subject to labor-market discrimination and their algorithmic representation. Previous research elaborates on the relation between the case workers' managerialism that “is fueled by competition and benchmarking” and the “responsibilization of citizen-customers” (Penz et al., 2017, p. 550). The primary responsibility of unemployment is shifted from the macro level of the labor market to the unemployed person that has to be activated in the counseling process and through active labor market programs (Glinsner et al., 2018, p. 2). Previously, the profiling of AMS customers relied on a situated evaluation of their competences, motivation and activities, and certainly case workers took into account characteristics, such as gender, age, ethnic descent, and condition of health. The algorithmic classification and prediction of job seekers' re-integration chances (IC scores) does not take into account aspects, such as soft skills and motivation, but produces a data-based numerical representation of the job seeker's individual risk of being subject to labor-market discrimination. Belonging to a group that is structurally discriminated leads to a deduction of this risk from the customers IC score. This objectified algorithmic representation of job seekers frames unemployment mostly in terms of the ‘employability' of individualized job seekers based on variables that represent their presumed structural discrimination on the labor market. In the same manner, variables, such as “level of education” and “occupational group” have high visibility in the system. “Employability” appears like a personal characteristic of a customer, which evokes neoliberal discourses that put the blame for not finding employment on the individual job seeker. At the same time, the limited agency of the job seeker to impact their IC score contradicts the neoliberal idea of an autonomous, “enterprising” individual (Rose, 1998) that has control over their success. Instead, the devised model has a tendency to essentialize “employability”—to conceptualize “employability” as something that is inherent to the individual, devoid of change, i.e., stable over time and contexts.

Moreover, the numerical representation of the risk of discrimination does not just depict the “harsh reality” of the labor market—another well-known neoliberal trope. This becomes particularly clear when considering the risk of being discriminated on the grounds of belonging to several disadvantaged groups. E.g., being a 51-years old women with a so-called ‘migration background' is represented in the model by simply adding the deductions for separate risk groups. This conceptualization is based on two assumptions: First, it presupposes that variables, such as gender, age or ethnic descent describe discrete groups that share a particular characteristic. However, one could hardly claim that all women have the same numerical risk of being discriminated given the diversity within the group in terms of e.g., education and social status and the diversity of social contexts, such as occupational groups. Second, it presupposes that discrimination on several grounds can simply be added up. However, critical race theory and feminist research have a long tradition of theorizing discrimination as intersectional (Crenshaw, 1989)^29^. In the context of labor market discrimination, intersectionality implies, e.g., that the experience of being a woman of Turkish descent cannot be understood in terms of being of Turkish descent and of being a women considered independently. Thus, being a female job seeker of Turkish descent may result in different forms of discrimination than being a man of Turkish descent or a woman without a so-called “migration background.” Hence, the AMS algorithm's tendency to essentialize the “employability” of customers in terms of their belonging to a structurally discriminated group re-enacts the activation paradigm in an ambivalent and differential manner.

Third, our analysis speaks to the coproduction of the algorithm and the “logic of workfare.” The study of Penz et al. (2017) reveals how case workers make up two categories of customers by differentiating between “good” and “bad” clients. The first category is perceived as pro-active job seekers, who do their best to find employment and thus act in accordance with the activation regime. Employment agents describe them as well-educated job seekers “who experience unemployment as a personal failure” (Penz et al., 2017, p.554). “Bad” customers on the contrary appear to have no intention of finding employment and are unwilling to comply with the rules of the integration contract. “People without vocational training (some with a migration background and language problems), with discontinuous work histories and little chances of reintegration regularly belong to this group.” (Penz et al., 2017, p.554) They do not obey the “reciprocity norm” (Serrano Pascual, 2007, p. 14) of the activation paradigm which constitutes benefit recipients as morally obliged to work. The authors of the study suggest, that the “distinction between these two groups also reflects the entrepreneurial spirit of employment agents” (Penz et al., 2017, p. 554). Interestingly, this distinction has also been implemented in the algorithmic system in terms of building different regression models for job seekers with continuous and with discontinuous work histories (with separate models for customers with or without a “migration background”). The distinction seems technically necessary due to the lack of data available on customers with discontinuous work histories, but this does not explain the differentiation between customers with or without a “migration background,” as argued earlier. Whether these design decisions will result in allocating fewer resources to customers with discontinuous work histories and/or clients with a “migration background” is not fully clear at this point in time. The AMS algorithm could either correct or reinforce the prejudiced categorization of job seekers into “good” and “bad” customers. However, our discussions of bias and discrimination in light of previous research in critical data studies and fairness in algorithmic systems gives reason to assume that the historical disadvantage is likely to become manifest in the system (Eubanks, 2018). Our analysis shows that the AMS algorithm re-enacts the activation paradigm by differentiating between citizens who get full support and citizens who are predicted to be long-term unemployed. In order to fulfill the “moral contract,” they have to participate in measures to “stabilize” and “motivate” them instead of having access to actual job training. Following the objective of raising the effectiveness of active labor market programs, allocating resources to group C would lower the performance of the individual case worker or the branch as compared to other local branches. In this way, the coincidence of previously established entrepreneurial techniques of the AMS and the technical data-requirements of the different regression models promotes the logic of workfare. The transformation of the welfare state to the workfare state sets wage labor as a necessary precondition for social participation and the autonomy of individuals. The objectified classification of group C clients presumably deprives them of the autonomy to have a say in what kind of support they do or do not require to find employment.

The coproduction framework “shows how certain conceptual designs and cognitive formulations gain ground at the expense of others, and how, once adopted, these successful settlements come to be seen as natural, inevitable, or determined in advance” (Jasanoff, 2006a, p. 277). The neoliberal adoption of the “market” as the organizing principle of politics and society may make it seem evident that a state's resources need to be allocated in an effective rather than in a just way. But contrary to the neoliberal framing of design decisions implemented in the Austrian system, countries, such as Australia, Ireland, and Sweden use statistical profiling to identify clients with high risk of long-term unemployment in order to provide privileged support for this group early on (Scoppetta et al., 2018). If (cumulative) discrimination on the labor market is taken seriously, structural measures need to be taken and respective resources appertain to the groups most disadvantaged.

As shown in detail in the previous section, there is a lack of transparency of many specifics of the AMS algorithm and its integration into the counseling process. In fact, only now (December 2019) central rules and guidelines for the implementation of the algorithm in AMS practices are negotiated and prepared for the roll-out that has just recently been postponed to July 2020. This poses challenges for an assessment of the effects that the system will develop and raises serious questions about the accountability of the system and its creators. However, our analysis of the AMS algorithm-in-the-making has shown that the profiling system is not a coherent technical artifact but that it produces tensions and ambivalences as part of everyday practices and broader political transformations. Further research is needed to analyze the algorithm-in-action “in collaboration with those facing them and perhaps understanding aspects that may not always be immediately apparent,” i.e., AMS case workers and customers (Veale et al., 2018, n.p.) once the algorithm has been rolled out all over Austria.

## Data Availability Statement

The datasets generated for this study are available on request to the corresponding author.

## Author Contributions

All authors contributed equally to this article and are listed in alphabetical order.

### Conflict of Interest

The authors declare that the research was conducted in the absence of any commercial or financial relationships that could be construed as a potential conflict of interest. The handling editor declared a past co-authorship with one of the authors AM.

## Footnotes

^1^The documents will be referred to by their respective acronyms throughout the rest of the text (i.e., “SYN 1” instead of Holl et al., 2018).

^2^All documents are written in German. The titles and quotes of the materials have been translated by the authors.

^3^orig. “Das AMS-Arbeitsmarktchancen-Modell.”

^4^In this work the term algorithm is not employed to refer to a strict technical definition commonly used in computer science, but instead as a synecdoche (Gillespie, 2014) for the whole system, including models, data and software implementation.

^5^“eAMS,” https://www.e-ams.at/eams-sfa-account/p/index.jsf.

^6^orig. “Hauptverband der Österreichischen Sozialversicherungsträger.”

^7^orig. “Arbeitsmarktservicegesetz.”

^8^orig. “Integrationschancen-Wert (IC).”

^9^German: Volksanwaltschaft.

^10^orig. “fragmentierter Erwerbsverlauf.”

^11^orig. “Migrationshintergrund.”

^12^orig. “ethische Herkunft” [sic!].

^13^Listed in SYN 1 are Vienna, Salzburg and Carinthia.

^14^See http://www.ilo.org/public/english/bureau/stat/isco/isco08/ as well as International Labour Organization (2019) for details.

^15^See https://www.ams.at/bis/bis/IscostrukturBaum.php.

^16^Armed Forces Occupations, Managers, Professionals, Technicians and Associate Professionals and Skilled Agricultural, Forestry and Fishery Workers.

^17^Specifically, Clerical Support Workers, Services and Sales Workers, Craft and Related Trades Workers and Plant and Machine Operators and Assemblers.

^18^The absolute number of individuals registered as job seekers peaked in 2017 at about 350,000 after a strong and steady rise since 2011. At that time there were slightly <250,000 job seekers (Arbeit Plus-Soziale Unternehmen Österreich, 2019).

^19^orig. “Besonderes Augenmerk muss hier auf die Erhöhung der Schulungseffektivität gelegt werden, damit nur jene Fördermaßnahmen zur Anwendung kommen, die auch tatsächlich für eine Integration in den Arbeitsmarkt tauglich sind.”

^20^orig. “Wenn die Evaluierung bislang tatsächlich ergeben hat, dass insbesondere schlechte Chancen korrekt prognostiziert wurden, so bedeutet das auch, dass im Zuge eines konkreten Geschäftsfalls vom AMS nicht adäquat auf dieses “schlechte Risiko” einer Person reagiert wurde: Die Betreuung des AMS hat laut dem vorliegenden Modell keine Verbesserung der Chancen für diese Personengruppe gebracht.”

^21^orig. “Stabilisierung der persönlichen Situation und Stärkung der Motivation.”

^22^orig. “um größtmögliche Objektivität bemüht im dem Sinne, dass die prognostizierte Integrationswahrscheinlichkeit …weitestgehend den realen Chancen am Arbeitsmarkt folgt.”

^23^orig. “Einschätzungen aber eher schlechter ausgefallen als von der Software berechnet.”

^24^orig. “empirisch schwer zu fassen.”

^25^orig. “laufend durchgeführte *Ex-post*-Evaluierungen [zeigten], dass die Treffsicherheit der individuellen Prognosen zwischen 80 und 85% läge.”

^26^orig. “Der zum Einsatz kommende Algorithmus sei nur als “technische Unterstützung” für die BeraterInnen des AMS gedacht; die Entscheidung im Betreuungsprozess hätten letztendlich die zuständigen BeraterInnen zu treffen.”

^27^orig. “Assistenzsystem ist, dass [sic!] ihnen nicht Verantwortung abnimmt, sondern hilft, dieser Verantwortung gerecht zu werden.”

^28^For an analysis of the gendering of public service work see Glinsner et al. (2018).

^29^The theory of intersectionality explains how overlapping or intersecting social identities relate to systems and structures of oppression and discrimination.
